# Psychological wellbeing and treatment adherence among cardio-renal syndrome patients in Yemen: a cross section study

**DOI:** 10.3389/fmed.2024.1439704

**Published:** 2025-01-07

**Authors:** Adel Omar Laradhi, Yan Shan, Mohamed Elsayed Allawy

**Affiliations:** ^1^School of Nursing and Health, Zhengzhou University, Zhengzhou, China; ^2^College of Nursing, University of Hail, Hail, Saudi Arabia; ^3^Department of Nursing Sciences, College of Applied Medical Sciences, Prince Sattam bin Abdulaziz University, Wadi Alddawasir, Saudi Arabia; ^4^Medical- Surgical Nursing Department, Faculty of Nursing Suez Canal University, Ismailia, Egypt

**Keywords:** cardiorenal syndrome, hemodialysis, psychological wellbeing, anxiety, depression, treatment adherence

## Abstract

**Background:**

Anxiety and depression are associated with adverse outcomes in cardiorenal syndrome patients undergoing hemodialysis, including decreased quality of life, poorer clinical parameters, and lower treatment adherence.

**Objective:**

This study aimed to examine the level of psychological wellbeing and its relationship with treatment adherence among dialysis patients with cardiorenal syndrome.

**Methods:**

This cross-sectional descriptive study was conducted between February and May 2021 on convenience sampling of 100 patients in two dialysis centers in Hadhramout, Yemen. Patients’ depression and anxiety levels were assessed using the Hospital Anxiety and Depression Scale (HADS)—Arabic version, and patient treatment adherence was assessed using the Treatment Adherence Questionnaire (TAQ). Descriptive statistics, Pearson’s correlation analysis, and multiple linear regression analyzes were performed to analyze data with a significance level set at *p* < 0.05.

**Results:**

The mean age ± standard deviation of participants was 53.46 ± 14.24 years. Most (90%) of patients had moderate to high levels of anxiety and depression. Most of the patients (87%) had a low level of treatment adherence. The findings revealed that psychological wellbeing is significantly association with treatment adherence *t* = 2.577 (95% CI 0.029, 0.225), *p =* 0.011.

**Conclusion:**

Anxiety and depression symptoms occurred more frequently among dialysis patients with cardiorenal syndrome, and there was a significant association between psychological wellbeing and treatment adherence. Our findings suggest that nurse managers should take into account that adding psychotherapies into the present cardiorenal syndrome treatment programs would improve patients’ clinical and psychological parameters and, consequently, their clinical outcomes while taking patient heterogeneity and resource limitations into consideration.

## 1 Introduction

Cardiorenal syndrome (CRS) is the term used to describe the bidirectional interaction between the heart and kidney in which failure of one organ leads to deterioration or pathological changes in the other (1). Based on the initial pathology, the Acute Dialysis Quality Initiative agreed on two main CRS categories, cardiorenal and reno-cardiac, and then further classified into five forms of CRS in 2008. As a result, the definition of CRS has become significantly more prominent (2, 3). CRS is classified into five types: type I, acute cardiorenal syndrome; type II, chronic cardiorenal syndrome; type III, acute renocardiac syndrome; type IV, chronic renocardiac syndrome; type V, secondary cardiorenal syndrome (4).

Many factors lead to changes in the heart, vascular, musculoskeletal, and respiratory systems in chronic kidney disease, which collectively compromise cardiovascular function (5). Cardiovascular disease (CVD) is the leading cause of morbidity and mortality among patients on hemodialysis (HD) for end-stage renal disease (ESRD) (6). More than 50% of dialysis patients have CVD, and HD patients have a relative risk of death from CVD events that is 20 times higher than that of the general population (7). Moreover, chronic renal dysfunction is present in approximately 40–50% of heart failure patients (8). The prevalence of end-stage renal disease in Yemen stands at 120 cases per million annually (9). The prevalence of chronic kidney disease (CKD) in Asia ranges from 7.0 to 34.3%, with an estimated 434.3 million individuals affected across the eastern, southern, and southeastern regions. The majority of this disease burden is concentrated in China and India, accounting for nearly 299.9 million cases (10). Research conducted from 2000 to 2016 indicated that the prevalence of chronic renal failure (CRF) stages 1–5 and stages 3–5 in Africa was 15.8 and 4.6%, respectively (11), while additional studies demonstrated that the prevalence of end-stage kidney disease in Middle Eastern countries, including Tunisia, Egypt, Turkey, and Iran, varied between 55 and 818 per million populations (12). Symptoms of depression and anxiety were more prevalent among dialysis patients (13, 14). Anxiety and depression have prevalence rates of 38 and 27%, respectively, among end-stage renal disease patients, according to a systematic review of 55 studies (15). Therefore, both cardiac and renal diseases should be managed effectively to prevent or reduce the development or worsening of the adverse effects.

Patients undergoing hemodialysis follow a highly complicated therapeutic regimen (16), adherence to which is essential to prevent problems from developing and to improve their survival (17). However, this regimen has a low compliance rate (18, 19). Therefore, early identification of poor treatment adherence and factors associated with it is critically important. Nevertheless, a variety of factors, including non-compliance to treatment, limited activities (20), financial difficulties (21), marital problems (22), co-morbidities, frequent hospitalizations (23), chronic pain, and sleep disturbances (24), may increase emotional distress and contribute to the development of anxiety and depression in hemodialysis patients.

Anxiety and depression have been associated with adverse outcomes in hemodialysis patients, including lower quality of life, worse clinical parameters (e.g., loss of vascular access, poorer nutritional status) (13, 25), lower treatment adherence (26, 27), increased risk for death or hospitalization (28–30), and more frequent suicidal ideation (31, 32). However, anxiety and depression continue to be under-recognized and neglected in patients with ESRD (33). At present, however, in Yemen, the relationship between the psychological wellbeing of CRS patients treated with dialysis and their treatment adherence has not been clarified. In addition, the rate of psychological wellbeing among Yemeni dialysis patients with CRS is unknown. Understanding this is crucial for administering appropriate treatment and care to such patients. The findings of this study highlight the importance of addressing the psychological aspects of patient care to improve treatment outcomes and overall health in Yemeni patients with CRS, illuminating the significant correlation between psychological wellbeing and treatment adherence.

## 2 Materials and methods

### 2.1 Design and setting

This is a descriptive cross-sectional study that was conducted in two dialysis centers in Hadhramout City, Yemen. Patients were enrolled during regular visits to the dialysis facilities from February to May 2021.

### 2.2 Procedure and participants

Criteria for enrollment were: 18-year-old or older Yemeni patients diagnosed with cardiorenal syndrome for at least 6 months. Participants had to be able to read and understand Arabic. Patients who have been receiving hemodialysis three times per week for at least 3 months prior to the study begin based on medical chart review. Patients who had a psychiatric or cognitive disease were excluded. All participants provided written consent that outlined the study’s goals, risks, and advantages. The participants were informed that their participation in this study was entirely voluntary and that they were free to withdraw at any time. Participants were informed that their anonymity would be thoroughly protected and that only aggregated data would be disclosed. Participants’ confidentiality was preserved. Face-to-face interviews were used to gather information from each participant. The interview took no more than 20 min to complete. Following the interview, the participant was asked to verify that their response was accurate. The interviewer received training about active listening, minimizing personal bias, and developing a well-structured interview guide prior to data collection.

### 2.3 Sample size and sampling method

Patients were enrolled in the study using a convenience sampling technique. The sample size was calculated using a single proportion formula at a 95% confidence level (Z score of 1.96) with a margin of error (E) of 0.1 and an estimated population proportion (p) of 0.5 (34). The initial calculated sample size was 97 participants. To account for a potential 5% non-response rate, the final sample size was adjusted to 100. The study samples were 100 patients with cardiorenal syndrome, attending dialysis centers during the study period and agreed to participate in the study.

### 2.4 Materials

The data were collected using three self-administered questionnaires:

#### 2.4.1 The sociodemographic and clinical characteristics

The researcher developed this instrument; the questionnaire has two sections: the first section has eight questions to assess participants’ sociodemographic characteristics, including age, gender, marital status, educational level, income, occupation, number of family members, and daily diet prepared. The second section has four questions to assess the patients’ clinical data.

#### 2.4.2 The hospital anxiety and depression scale

The hospital anxiety and depression scale (HADS) is a 14-item self-report questionnaire used to measure an individual’s levels of depression (7 items) and anxiety (7 items) over the past week, which have a 4-point Likert scale (from 0 to 3). Maximum scores for each subscale are 21, and the scores in each subscale are computed by summing the corresponding items. A score of 0–7 is considered normal, 8–10 is considered borderline, while 11–21 is considered a case (anxiety or depression). This tool was developed in Arabic by Terkawi et al. (35), and the Cronbach’s alpha for the Arabic version of HADS is 0.84, which can be regarded as an excellent value (35).

#### 2.4.3 The treatment adherence questionnaire

The Treatment Adherence Questionnaire (TAQ) was used to evaluate treatment adherence in patients with CRS dependence on dialysis. This instrument includes dialysis adherence, medication adherence, fluid restriction adherence, and diet restriction adherence. The questionnaire contained 15 items, and the response options ranged from 1 to 4 on a 4-point Likert scale (1 = never, 2 = sometimes, 3 = most of the time, 4 = all the time). The total treatment adherence scores were computed by summing up the scores from items 1 to 15, after reverse scored items 2, 3, 6, 8, 10, 14, and 15. The range of possible (or potential) total scores is 15 to 60. A better TAQ score indicates better treatment adherence. The total scores are categorized into three: 15–29 (<50%) indicated a low level of treatment adherence, 15–29 scores indicate low levels of treatment adherence; 30–44 scores indicate moderate levels of treatment adherence; and 45–60 scores indicate a high level of treatment adherence (36). The questionnaire was adopted from a previous study by Kritpracha (37), and the Cronbach’s alpha for the TAQ was 0.827, which can be considered an excellent value, which supports the internal consistency reliability of this instrument (37).

### 2.5 Data analysis

The data were cleaned to remove all errors from the data collection. The demographic data and health information of the study participants with CRS receiving dialysis are presented using descriptive statistics, which include mean, standard deviation, frequency, percentage, range, and minimum and maximum values. The relation between psychological wellbeing and treatment adherence behavior was assessed using linear regression analysis and bivariate Pearson correlation analysis, and differences between groups were analyzed using one-way ANOVA. Data were analyzed using the program SPSS 23.0 for Windows (SPSS, Inc., Chicago, IL) with statistical significance set at *p* < 0.05.

## 3 Results

### 3.1 Patients’ characteristics

Among 100 patients diagnosed with cardio-renal syndrome, 63% of patients were male, and 73% were older than 40 years. Patients had a mean age of 53.46 (SD = 14.24). Most patients were married (97%) and had insufficient monthly income (99%). The majority of the patients (80%) were diagnosed with cardio-renal syndrome stage 4. The majority of the patients (68%) had hypertension disease. Table 1 shows the patients sociodemographic and clinical characteristics in more detail.

**Table 1:** Sociodemographic and clinical characteristics (*N* = 100).

Characteristics variable	Frequencies (N)	Percentage (%)	(Mean ± SD)
**Gender**			
Male	63	63	
Female	37	37	
**Age (years)**			(53.46 ± 14.24)
40 or older	73	73	
Less than 40	27	27	
**Monthly income**			
Enough	1	1	
Not enough	99	99	
**Marital status**			
Not married	3	3	
Married	97	97	
**Disease stage**			
Stage I	4	4	
Stage II	12	12	
Stage III	4	4	
Stage IV	80	80	
**Education level**			
No formal education	34	34	
Primary school	41	41	
Secondary school	15	15	
Tertiary education or above	10	10	
**Occupation**			
Full-time	20	20	
Part-time	8	8	
Retired	6	6	
Unemployed	66	66	
**Number of family number**			
Two person	17	17	
More than two	83	83	
**Diet prepared by**			
Patient	3	3	
Family member	95	95	
Other	2	2	
**Length of diagnosis with CRS**			
Less than 5	73	73	
5–10 years	18	18	
More than 10	9	9	
**Length of hemodialysis**			
Less than 5	56	56	
5–10 years	25	25	
More than 10	19	19	
**Comorbidities**			
Diabetes mellitus	23	23	
Hypertension	68	68	
Other	9	9	

### 3.2 Anxiety and depression level

#### 3.2.1 Anxiety level

The total anxiety mean score among 100 CRS patients was 1.61 ± 0.63, and 90% of the participants reported moderate to high levels of anxiety (Table 2).

**Table 2:** Status of psychological wellbeing and two dimensions (*N* = 100).

Dimensions	Low (normal) *n* (%)	Moderate *n* (%)	High *n* (%)
Anxiety	10	60	30
Depression	20	75	5
Total	10	65	25
Mean ± SD of Anxiety = 1.61 ± 0.63			
Mean ± SD of Depression = 1.23 ± 0.78			

#### 3.2.2 Depression level

The total depression mean score among 100 CRS patients was 1.23 ± 0.78, and 80% of the participants reported moderate to high levels of depression (Table 2).

#### 3.2.3 Anxiety and depression level

The degree of anxiety and depression was 25% of patients reported high anxiety and depression, whereas 65% had moderate levels of both. Another way to put it was that 90% of the patients experienced moderate to high levels of anxiety and depression (Table 2).

### 3.3 Treatment adherence level

The overall treatment adherence mean score among 100 CRS patients was 2.73 ± 0.22. The level of treatment adherence was analyzed as shown in Table 3. Most of the patients (87%) had low levels of treatment adherence. Regarding each dimension of treatment adherence, the dimensions of adherence to medication and diet restriction were performed at a low level. Other dimensions, adherence to hemodialysis and adherence to fluid restriction, were at a moderate level.

**Table 3:** Status of treatment adherence and four dimensions (*N* = 100).

Treatment adherence dimensions	Low *n* (%)	Moderate *n* (%)	High *n* (%)
Adherence to hemodialysis	2	90	8
Adherence to medication	85	5	10
Adherence to fluid restriction	22	67	11
Adherence to diet restriction	87	12	1
Total	87	12	1
Mean ± SD = 2.73 ± 0.22			

*Adherence interpretation: High = 45–60, Moderate = 30–44, Low = 15–29.

### 3.4 Relationship between psychological wellbeing and treatment adherence

Psychological wellbeing is significantly association with treatment adherence. *t* = 2.577 (95% CI 0.029, 0.225), *p* = 0.011 (Table 4).

**Table 4:** Linear regression analysis between psychological wellbeing and their total adherence (*N* = 100).

Variable	Treatment adherence			
	*t*	95% CI		*p* value
Psychological wellbeing	2.577	0.029	0.225	0.011

## 4 Discussion

This was the first study in Yemen to examine the psychological wellbeing among hemodialysis patients with CRS and explore the relationship between psychological wellbeing and treatment adherence among hemodialysis patients with CRS. In the present study, results showed that 90% of the patients experienced moderate to high levels of anxiety and depression. Regarding the level of anxiety, 60% of patients had moderate levels, and 30% had high levels of anxiety and concern to depression dimension, 75% of patients had moderate levels of depression, and 5% had high levels of depression. Prior research has shown that prevalence varies by population and measurement tools (ranging from 22.5% to nearly 85% for depression and from 32.3 to 44.7% for anxiety) (25, 38, 39) as well as the range of the current global prevalence estimates for the general population (3.7–48.3%) (40). However, in a meta-analysis, the prevalence was higher than that of individuals with end-stage renal disease or chronic kidney disease (26.5%) (41). Anxiety and depression may be normal responses to these situations in hemodialysis patients, as patients undergoing hemodialysis exhibit psychophysiological symptoms related to the effect of disease on the occupational, social, physical, and personal aspects of patients’ lives. This finding is consistent with the results of previous studies, which concluded that depression and anxiety symptoms were more prevalent among dialysis patients (42, 43). Moreover, other studies found that patients on chronic dialysis are at high risk of anxiety and depression (44–46).

While the majority of participants with cardiorenal syndrome were male. This study demonstrated that there were statistical differences in the level of depression and anxiety scores and gender of patients, and females had a higher mean score for anxiety and depression than males. A linear regression study with a regression coefficient (*β*) of 5.58 and a statistically significant *p*-value of 0.004 found that gender explains 8.9% of the difference in anxiety and depression ratings, while other demographic factors like age, marital status, income, employment, and education did not significantly affect overall anxiety and depression levels or increase the explained variation when incorporated into the regression model. The findings are in line with a study that showed women show more depression and anxiety symptoms as compared to men (47) and inconsistent with another study that concluded that men show more depression and anxiety as compared to women (48). This implies that, despite the significant influence of gender on anxiety and depression, the inclusion of these other variables did not improve our understanding of the mechanisms that influence anxiety and depression levels, prompting future investigation of additional predictors.

Recent studies have emphasized the importance of psychological variables in the development and progression of coronary heart disease (CHD). For example, a systematic review offers a thorough examination of the association between psychological variables and CHD. It emphasizes the critical role that depression and anxiety play in the development and progression of CHD, encouraging healthcare practitioners to consider psychological issues as significant risk factors for the condition (49, 50). Furthermore, previous studies have shown that psychological disorders such as depression may have a causal relationship with an increased risk of HF (51, 52). However, mental disorders can influence the development of HF through a variety of mechanisms. First, the hyperactivation of the sympathetic nervous system in individuals with mental problems may facilitate the development of HF. Studies have linked elevated serum adrenaline levels to anxiety disorders, depression, and sleeplessness (53–55). Furthermore, compared to healthy controls, participants with untreated bipolar disorder had higher serum levels of the noradrenaline metabolite (56). Antipsychotics can also raise catecholamine levels in the plasma (57). This causes the heart’s sympathetic nerve signals to stay high all the time, which may help HF develop (58). Secondly, reduced left ventricular ejection fraction and cardiac remodeling can result in HF (59). This may be caused by the inherent effects of mental diseases, the poor lifestyle habits of individuals with mental disorders, and the effects of antipsychotic medication (59, 60). Therefore, it is important that patients with cardiorenal syndrome, especially those on hemodialysis, have increased screening for psychological issues and receive enough care from healthcare providers.

The present study showed a linear regression and significant association between psychological wellbeing and treatment adherence, indicating that patients’ good psychological wellbeing may be positively affecting patients’ adherence to treatment regimens. Our finding is consistent with previous studies on which anxiety and depression have been linked to poorer clinical parameters among patients on hemodialysis (e.g., poorer nutritional status) (25) and reduced treatment adherence (27, 61). In this regard, our findings disclose many variables (clinical, psychological, cognitive, and behavioral) surrounding the treatment of cardiorenal syndrome patients. The two most common psychological problems, such as anxiety and depression, influence treatment adherence among patients with cardiorenal syndrome undergoing hemodialysis. Therefore, this implies nurse administrators should consider the development of novel therapeutic approaches in this field. Based on our findings, we suggest that nurse administrators should consider that incorporating psychotherapies into the current cardiorenal syndrome treatment programs would improve patients’ clinical and psychological parameters and, consequently, their clinical outcomes.

## 5 Strengths and limitations

The strength of this study is that, to our knowledge, it is the first study to assess different aspects of psychological wellbeing in a considerable number of CRS patients treated with dialysis in Yemen and to identify the relationship between psychological wellbeing and treatment adherence. Despite its strength, there are some limitations: First, all the participants in this study were Muslims or from one area (Hadhramout Province) with no significant number of other religions or other regions, making it difficult to generalize the results to other parts of Yemen or other nations or religions. Second, inadequate sample size due to decreased patient flow during the COVID-19 pandemic period. Third, we did not assess those patients who used antidepressant medications without a definite diagnosis by a physician. Lastly, a convenience sampling method was one of the limitations of this study, and due to the cross-sectional design, causal relationships could not be established, nor could the progression of anxiety and depression symptoms over time be measured.

### 5.1 Implications for the profession and/or patient care

In clinical settings, our findings suggest that nurse administrators should consider that incorporating psychotherapies into the current cardiorenal syndrome treatment programs would improve patients’ clinical and psychological parameters and, consequently, their clinical outcomes. The two most common psychological problems, such as anxiety and depression, influence treatment adherence among patients with CRS undergoing hemodialysis. Therefore, this implies nurse administrators should consider the development of novel therapeutic approaches in this field. Although the effect of gender is considerable, the low percentage of explained variance indicates that other factors likely influence anxiety and depression levels in this cohort. Therefore, this suggests that further research should be conducted to identify additional predictors.

## 6 Conclusion

Our study showed that most patients had moderate to high levels of anxiety and depression. Most of the patients had low levels of treatment adherence. This study revealed a linear regression and significant association between psychological wellbeing and treatment adherence. Based on our findings, it is suggested to incorporate psychological treatment to improve patients’ clinical and psychological parameters and, consequently, their adherence to the treatment regimen. While taking patient heterogeneity and resource limitations into account, further research is required to develop a psychotherapeutic program and to evaluate its effect based on illness perception or patient understanding on reducing anxiety and depressive symptoms in cardiorenal syndrome patients undergoing hemodialysis.

## Data Availability

The original contributions presented in the study are included in the article/supplementary material, further inquiries can be directed to the corresponding author.
